# P05.35. What are participants in clinical trials told about placebos? A content analysis of participant information leaflets

**DOI:** 10.1186/1472-6882-12-S1-P395

**Published:** 2012-06-12

**Authors:** F Bishop, A Adams, T Kaptchuk, G Lewith

**Affiliations:** 1 University of Southampton, Aldermoor Health Centre, Southampton, England; 2 Northern Arizona University, Flagstaff, USA; 3 Harvard University, Cambridge, USA

## Purpose

To identify what participants in major RCTs in the UK are told about placebos and their effects.

## Methods

The major registry of current clinical trials in the UK (UKCRN) was searched to identify trials conducted in clinical populations using placebo controls. Emails were sent to 182 contact personnel requesting they send their participant information leaflets (PILs) for inclusion in the study. Forty-nine PILs were received; 45 were included in the analysis (4 were ineligible). Qualitative and quantitative techniques of content analysis were used to identify characteristics of the trials, how the placebo and target treatment were explained, the presence or absence of information about possible effects of the target and placebo treatments, and options concerning un-blinding and possible treatments after the trial.

## Results

Placebos and target treatments were described quite differently. In almost every comparison, the target treatments were prioritized over the placebo, from the words in the title to the description of what would happen at the end of the trial. The placebo was described as a scientific tool that would allow efficacy of the target treatment to be determined; the target treatment was described as a treatment that might generate health effects. Placebo treatments were referred to less frequently than target treatments and were significantly less likely to be described as triggering either positive or adverse health effects (p<.01). A minority of PILs mentioned un-blinding or treatment options after the trial, and the focus of the latter was primarily on the possibility of continuing with the target treatment.

## Conclusion

Trial participants were poorly informed about the health changes that they might experience if they were allocated to a placebo treatment, and of options that might be available to them after the trial. Different ways of describing placebos to participants, in PILs and in person, should be developed and tested.

